# MRI characteristics and correlation with pathology of an unusual inflammatory myofibroblastic tumor of the bladder

**DOI:** 10.1259/bjrcr.20220086

**Published:** 2022-11-01

**Authors:** Wanling Ma, Xu Huang, Nanai Xie, Qi Tan, Heng Zhang, Yuhui Zhu, Xingzhi Li

**Affiliations:** 1 Department of Radiology, The Second Affiliated Hospital, School of Medicine, The Chinese University of Hong Kong, Shenzhen, Guangdong Province, 518172, China; 2 Department of Pathology, The Second Affiliated Hospital, School of Medicine, The Chinese University of Hong Kong, Shenzhen, Guangdong Province, 518172, China; 3 Department of Urology, The Second Affiliated Hospital, School of Medicine, The Chinese University of Hong Kong, Shenzhen, Guangdong Province, 518172, China

## Abstract

We report a case of inflammatory myofibroblastic tumor of the bladder (IMTB) that arises from left posterior bladder wall. The IMTB usually demonstrates slight hypointensity on T1WI, heterogeneous bright hyperintensity on T2WI, hyperintensity on DWI, and no restricted diffusion on ADC map. IMTB exhibits irregular ring enhancement and scatters stripe-like enhancement in central area with progressive and persistent enhancement pattern on CE-MRI. High-contrast multisequence MRI may be a potential technique to distinguish IMTB from other bladder tumors.

## Introduction

Inflammatory myofibroblastic tumors (IMTs) are rare benign spindle cell tumors and characterized histologically by the myofibroblastic spindle cell proliferation accompanied by inflammatory cell infiltration.^1^ Although IMTs most commonly occur in the lung, they can also present in diverse extrapulmonary locations including head and neck, abdomen, pelvic cavity, retroperitoneum, trunk, and limbs.^2^ IMTs arising from the urinary system are relatively rare and mostly locate in the bladder.^3^ IMTs of the bladder (IMTB) are typically considered as benign; however, recurrences have been reported in the literatures.^3^ The diagnosis of IMTB may be delayed by non-specific clinical symptoms and laboratory tests. Therefore, preoperative identification of IMTB by imaging technique is very important for deploying the individually tailored therapies.

To our knowledge, there has been few reports focused on the correlation between imaging and pathological features. Herein, we present a case report regarding MRI characteristics of a rare IMTB and its relationship to pathological features.

## Case report

A 26-year-old female patient was admitted to the emergency department due to gross hematuria accompanied by urinary urgency and frequency for half a day. Routine ultrasound showed a pelvic mass. Physical examination on admission revealed no suprapubic pain or palpable abdominal mass. Routine blood examination indicated anemia and urinalysis showed an increased number of homogeneous red blood cells. Other laboratory results were unremarkable.

**Figure 1.**
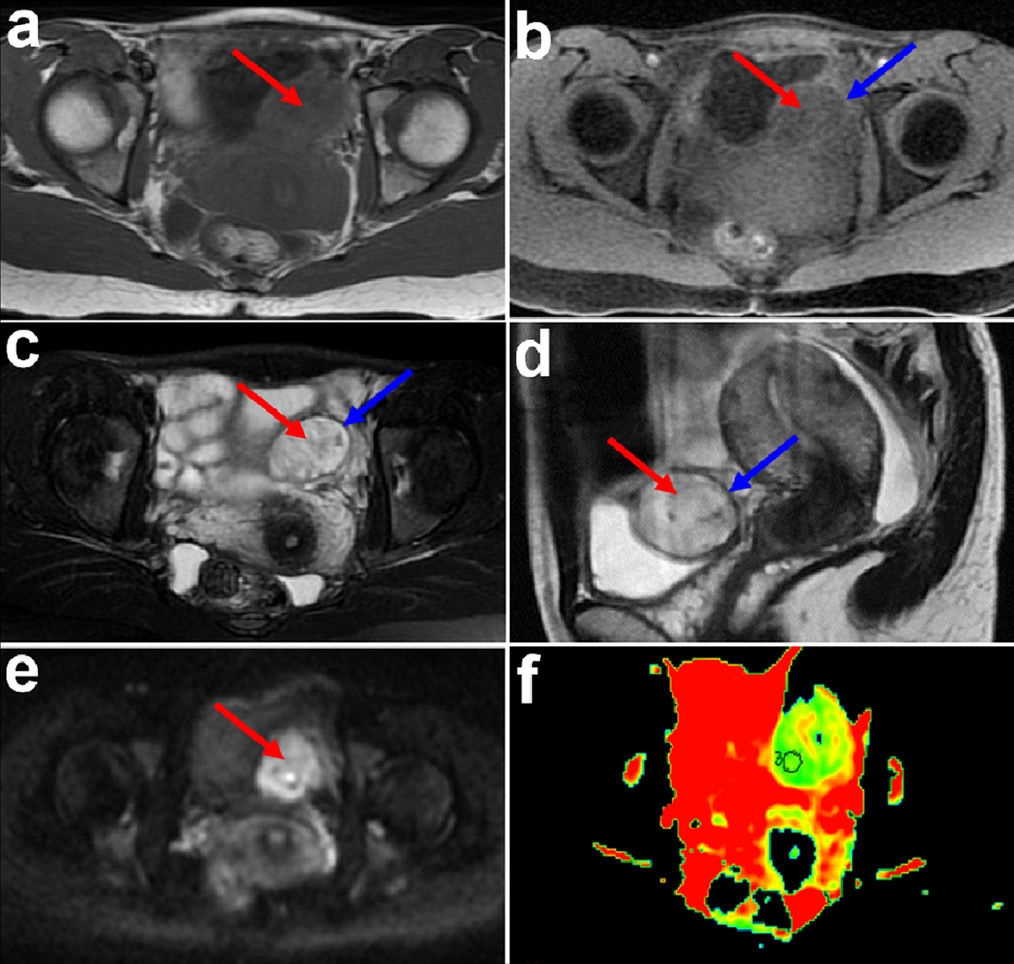
MRI and DWI of the IMT locating in the bladder. (**a**) Axial T1WI shows a well-defined, oval-shaped extravesical mass (58 × 25×13 mm) adhering to the left posterior bladder wall and protruding through the bladder lumen with slight hyperintensity compared to muscle (red arrow). (**b**) Fat-saturated axial T1WI exhibits hypointensity mass (red arrow) with isointensity capsule (blue arrow) compared to muscle. (**c, d**) Axial T2WI with fat-suppression(c) and sagittal *T*_2_-weighted images without fat-suppression (**d**) show the lesion with an isointensity rim (blue arrow) and bright hyperintensity center (red arrow) to muscle. (**e**) Axial DWI (*b* = 800 sec/mm^2^) exhibits heterogeneous significant high signal-intensity (red arrow). (**f**) ADC values is 1.55 × 10^−3^ mm^2^/s. ADC, apparent diffusion coefficient; DWI, diffusion-weighted imaging; MRI, magnetic resonance imaging; T1WI, *T*_1_-weighted image; T2WI, *T*_2_-weighted images

**Figure 2.**
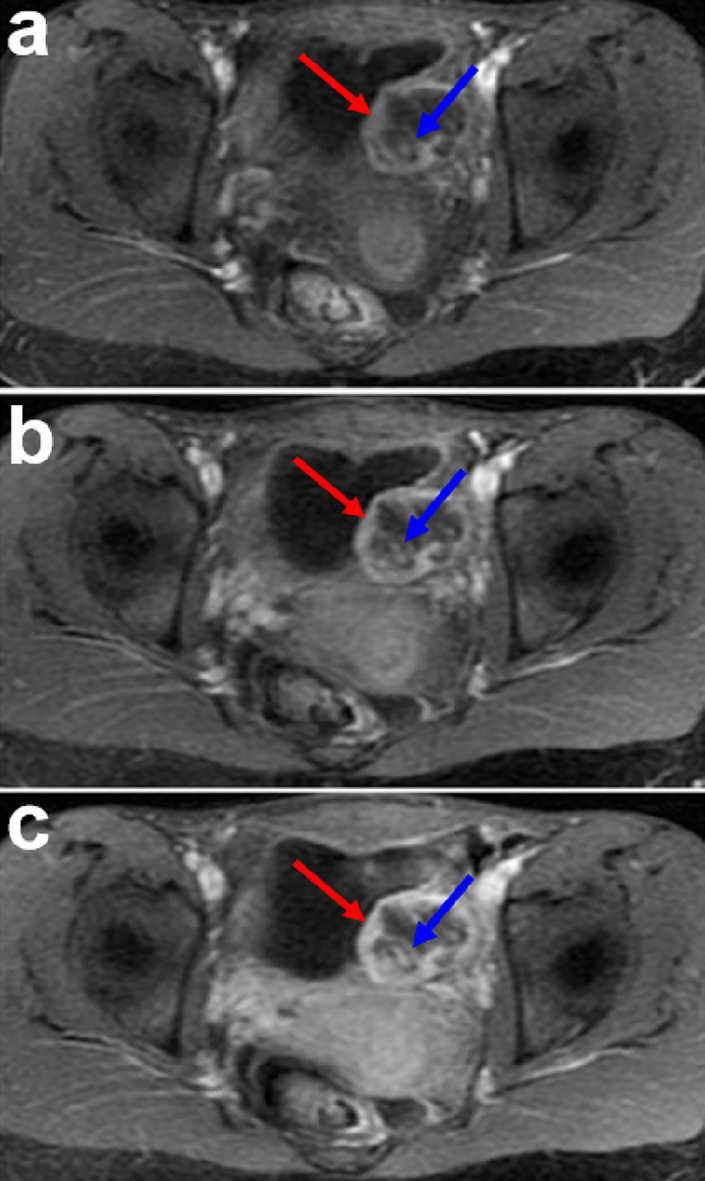
Contrast enhanced MRI of the IMT. (**a-c**) Axial gadolinium-enhanced fat-suppressed *T*_1_-weighted MR image on arterial phase(a), venous phase(b) and delayed phase (**c**) images show irregular ring enhancement in the peripheral area (red arrow) and scattered stripe-like enhancement in the central area (blue arrow) with progressive and persistent enhancement pattern. IMT, inflammatory myofibroblastic tumor; MRI, magnetic resonance imaging

Pelvis MRI revealed a well-defined, oval-shaped extravesical mass (58 × 25 × 13 mm) adhering to the left posterior bladder wall and protruding through the bladder lumen. The central area of the mass exhibited slight hyperintensity on axial *T*_1_-weighted images (T1WI) and mild hypointensity on T1WI with fat-suppression compared with muscle (Figure 1a–1b), heterogeneous bright hyperintensity on axial *T*_2_-weighted images (T2WI) with fat-suppression and sagittal T2WI without fat-suppression (Figure 1c–1d). The mass exhibited isointensity to muscle on both T1WI and T2WI in the peripheral area (Figure 1b–1d). The mass exhibited hyperintensity on diffusion-weighted imaging (DWI) (Figure 1e). Apparent diffusion coefficient (ADC) value is 1.55 × 10^−3^ mm^2^/s on ADC map (Figure 1f). On T1WI with contrast arterial phase image, the lesion exhibited irregular ring enhancement with relatively well-defined margin and scattered stripe-like enhancement in the central area (Figure 2a). The mass exhibited progressive and persistent enhancement on venous phase and delayed phase images (Figure 2b–2c).

**Figure 3.**
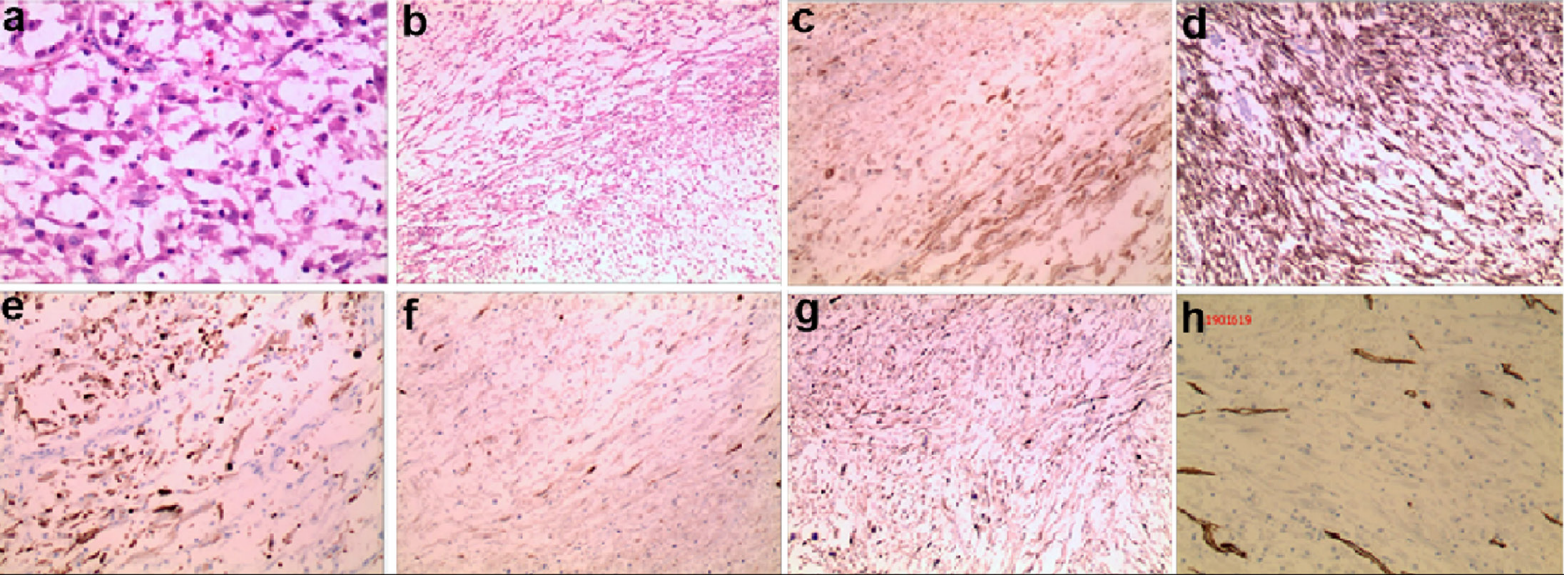
Histopathologic images of the IMT. (**a**) H&E staining (400×) exhibits stellate-to-plump spindle cells in an oedematous and myxoid background. (**b**) H&E staining (10×) exhibits the higher cellularity regions. (**c-h**) Immunohistochemical staining exhibits that the tumor cells are positive for Vimentin, ALK, CK, SMA, Desmin, and Ki-67 Index respectively. ALK, anaplastic lymphoma kinase; CK, cytokeratin; IMT, inflammatory myofibroblastic tumor; SMA, smooth muscle actin

The patient subsequently underwent the bladder mass resection. The specimens were collected for pathologic evaluation and results were shown in Figure 3a–3h. The tumor was composed of spindle cell tumor with an abundant edematous and myxoid background. Inflammatory cells (mainly lymphocytes) were scattered in the myxoid background. Immunohistochemical analysis manifested that the tumor cells were positive for smooth muscle actin (SMA), Vimentin, Desmin, anaplastic lymphoma kinase (ALK) and cytokeratin (CK), but negative for CD-34, indicating a consistent diagnosis of IMTB. The Ki-67 Index was 5%, indicating the high cellular proliferative activity.

## Discussion

IMTB is an extremely rare tumor and barely diagnosed in children.^4^ Most IMTB patients are young adults and females are more susceptilbe than males (ratio 5:3).^3,5^ Hematuria is the most common clinical manifestation of IMTB that occurs in 81.3% of patients.^5^ IMTB commonly occurs in the superior wall or front wall of the bladder.^6^ IMTB usually exhibits the spindle cell proliferation and inflammatory cell infiltration, while lacks of significant necrosis, abnormal mitotic figures and cytologic atypia.^7^ The local tumor recurrence rate of IMTB after complete resection is only 4% and few distant metastases have been reported.^3^

IMTB can be large with a mean size of 5.5 cm due to their specific location and slow growth. MRI features of IMTB have diverse manifestations depending on the contents of mucus, fibrosis and cellular infiltration. In the central part of IMTB as our case, it may appear isointensity or slight hyperintensity on T1WI and heterogeneous bright hyperintensity on T2WI which correlates with a few spindle cells and inflammatory cells scattered in the abundant edematous mucus background. Isointensity on both T1WI and T2WI in the peripheral area can be interpreted as compact arranged spindle cells with scattered chronic inflammatory cells and many blood vessels which result in more obvious and progressive enhancement after contrast.^6^ As previous studies^5,6^ reported that necrosis or cyst degeneration was rare in IMTB, there was no necrosis or cyst degeneration occurred in our case. Because the MRI features of IMTB are diverse depending on the pathological component, histopathology assessment may provide the good explanation for MRI findings of IMTB. Immunohistochemical positive findings including Vimentin, Desmin, SMA, S-100 protein and negative for CD-34 can confirm the definitive diagnosis of IMTs.^8^ Approximately 35–89% of IMTB presents with ALK-1 over-expression.^3^

IMTB is generally considered to be benign with few distant metastases being reported to date. It is essential for urologist to get a correct diagnosis in order to avoid unnecessary radical surgery of bladder. Therefore, it is very important to differentiate IMTB from bladder malignant tumors such as transitional cell carcinoma (TCC) and rhabdomyosarcoma. Bladder TCC often occurs in male older than 65 years and usually occurs in the base of bladder.^9^ However, IMTB most commonly occurs in young adults and most of them locate in the superior wall and front wall with no case in the bladder base.^3,5,6,9^ After contrast, bladder TCC usually demonstrates homogeneous or heterogeneous enhancement with peak appearing after a delay of approximately 60 sec on DCE-MRI. However, IMTB often displays ring enhancement type which may be a clue for diagnosing IMTB on DCE-MRI.^5^ Bladder TCC usually presents obvious restricted diffusion on DWI due to its high cellularity. However, no diffusion restriction occurs in IMTB because of its abundant edematous mucus background like our case. The hyperintensity of IMTB on DWI in our case can be interpreted as T2 “shine-through” effects. Bladder rhabdomyosarcomas are the most commonly-occurring solid neoplasms in children younger than 10 years^9^. Bladder rhabdomyosarcomas most often locate in vesical trigone and bladder neck and appear as the large and nodular heterogeneous enhancement mass with evident necrosis on contrast-enhanced MRI.^9^ However, IMTB usually locates in the superior or front wall and appears as ring-shaped enhanced masses without evident necrosis.^6^ Bladder leiomyomas are rare benign tumors without risk of recurrence or metastasis. Their MR imaging features are very similar to uterine fibroids which demonstrate intermediate to low signal intensity on both T1WI and T2WI.^10^ Because IMTB has the potential of recurrence and about 60% of it has the invasion into the muscularis propria,^3^ transurethral resection of bladder tumor or partial cystectomy is recommended to prevent any recurrence. Therefore, it is important to differentiate IMTB from bladder leiomyomas before surgery to avoid inappropriate treatment. Different from bladder leiomyomas, IMTB usually demonstrates hypointensity on T1WI and heterogeneous bright hyperintensity on T2WI due to its abundant edematous mucus. Different MRI features can distinguish IMTB from bladder TCC, rhabdomyosarcoma, and leiomyomas.

## Conclusion

In conclusion, IMTB usually demonstrates hypointensity on T1WI, bright hyperintensity on T2WI, irregular ring-shaped progressive enhancement on contrast-enhanced MRI, and without diffusion restriction on ADC map. High-contrast multisequence MR imaging can accurately distinguish IMTB from other bladder tumors by identifying these MRI characteristic findings.

## Learning points

- IMTB usually appears slight hypointensity on T1WI, heterogeneous bright hyperintensity on T2WI with fat-suppression in the central area, and the mass exhibits isointensity to muscle on both T1WI and T2WI in the peripheral area.
- IMTB exhibits irregular ring enhancement with relatively well-defined margin and scattered stripe-like enhancement in the central area on contrast arterial phase image, and the mass exhibits progressive and persistent enhancement on venous and delayed phase images.
- IMTB exhibits hyperintensity on DWI due to T2 “shine-through” effects and no diffusion restriction on ADC map.
